# Factors Associated with Fear of Falling among Community-Dwelling Older Adults in the Shih-Pai Study in Taiwan

**DOI:** 10.1371/journal.pone.0150612

**Published:** 2016-03-02

**Authors:** Hsiao-Ting Chang, Hsi-Chung Chen, Pesus Chou

**Affiliations:** 1 Department of Family Medicine, Taipei Veterans General Hospital, Taipei, Taiwan; 2 Institute of Public Health and Community Medicine Research Center, National Yang-Ming University, Taipei, Taiwan; 3 School of Medicine, National Yang-Ming University, Taipei, Taiwan; 4 Department of Psychiatry & Center of Sleep Disorders, National Taiwan University Hospital, Taipei, Taiwan; San Francisco Coordinating Center, UNITED STATES

## Abstract

**Background:**

Fear of falling is an important risk indicator for adverse health related outcomes in older adults. However, factors associated with fear of falling among community-dwelling older adults are not well-explored.

**Objectives:**

To explore the quality of life and associated factors in fear of falling among older people in the Shih-Pai area in Taiwan.

**Methods:**

This community-based survey recruited three thousand eight hundred and twenty-four older adults aged ≥ 65 years. The measurements included a structured questionnaire, including quality of life by using Short-Form 36, and information of fear of falling, fall history, demographics, medical conditions, insomnia, sleep quality, depression and subjective health through face-to-face interviews.

**Results:**

A total of 53.4% of participants reported a fear of falling. The rate of fear of falling was higher in female subjects. Subjects with fear of falling had lower Short Form-36 scores both for men and women. Falls in the previous year, older age, insomnia, depression and worse subjective health were correlates of fear of falling for both sexes. Male-specific associations with fear of falling were the accessibility of medical help in an emergency, diabetes mellitus and stroke. In parallel, cardiovascular diseases were a female-specific correlate for fear of falling.

**Conclusions:**

Fear of falling is prevalent among community-dwelling older adults. It is seems that there are gender differences in fear of falling with respect to the prevalence and associated factors in older adults. Gender differences should be considered when planning prevention and intervention strategies for fear of falling among older people.

## Introduction

Fear of falling (FOF) is a common problem in older adults living in the community [1,2] and has a prevalence ranging from 20% to 85% [3–13]. Initially, FOF was considered as post-fall syndrome [14], named ptophobia [15], however, subsequent studies found that FOF could also be identified in older adults who have not fallen [1,2,16–18]. FOF is reported to be associated with several adverse consequences, including mobility or activity restriction and the development of deconditioning [4,6,19–21], reduced social interactions [1,3,18], subsequent falls [2,22–24] and a poor quality of life which was found to be related to further physical, psychological and mental function declines [18,24].

Previous studies reported that older age, being female [1,2,4,9,10,25], history of previous falls [4,7,9,10], impaired physical performance [11,25,26], depressive disorders [7,9,25,27,28], impaired cognitive function [11–13,25,28–30], living alone [6,28] or having fewer social contacts [1], poor subjective health [2,4,10,25], lower educational level [10] or illiterate [7], having chronic health conditions [7,10] were relevant risk factors of FOF among older adults living in the community. Interventions including physical training [29,31–34], cognitive training [29,35], and guided relaxation and imagery therapy [36] have revealed effects on decreasing FOF among older people under different conditions. A recent study in Korea found that the multifactorial fall prevention program including exercise, education and general physical therapy is effective in improving FOF in post-stroke inpatients [37]. Although many domains of risk factors for FOF in older adults have been explored, there are still other important aspects that remain under-investigated, such as medical help accessibility which could be imperative for evaluation and prevention of FOF in older adults.

Among community-dwelling older adults, women had a higher prevalence of FOF than men [1,3,9]. Therefore, several previous studies focused on exploring the impact and risk correlates of FOF in older women. Predisposing factors for older women to develop FOF include advanced age, visual impairment, a sedentary lifestyle, and no available emotional support [22]. In addition, another study reported neuroticism, a history of falling, experience of fractures, a need to use both arms to push up to rise from a chair, poor subjective general health and living alone were associated with FOF in community-dwelling women aged over 70 years [38]. In contrast, how FOF affects quality of life in older men and whether the pattern of associated factors of FOF is comparable in both sexes are unknown.

In sum, although previous studies have illustrated that older adults with FOF had a poor quality of life, no studies compares the pattern of impact with respect to different sexes. Furthermore, factors associated with FOF have not been well investigated in older men. Thus, using a large cohort in Shih-Pai area in Taiwan, this study aimed to examine quality of life among community-dwelling older adults who reported FOF, and identify correlates of FOF and their respect gender-specific pattern of FOF among older adults residing in community.

## Methods

### Study Design, Setting and Subjects

This study was a part of the Shih-Pai Study in Taipei, Taiwan. Potential eligible participants were identified from the government household registration system. According to the 1999 official resident registration database in Taipei, there were 9,141 residents aged ≥ 65 years living in the Shih-Pai area. Among these residents, 523 were living in the long term care facilities, 175 died before an interview was conducted, and there were 1,292 vacant households. Residents who lived in the long term care facilities or had a diagnosis of dementia or severe cognitive impairment were excluded. After door-to-door face-to-face interviews, 1,255 potential eligible subjects were not reached for 3 visits, and 1,832 refused to be interviewed. Thus, 4,064 subjects accepted the face-to-face interview and 3,824 completed the FOF interview process during 1999 to 2002. The response rate was 53.5%. This study was approved by the institutional review board of the Taipei Veterans General Hospital in Taiwan. All participants provided written informed consent which was approved by the institutional review board of Taipei Veterans General Hospital before participation.

### Measurements

#### Fear of Falling and History of Falls

Two fall-related questions were recorded. The first was “Are you presently afraid of falling?” This question has been used in previous studies [2,22,23] with good test-retest reliability [23,39]. The second question, related to a history of falls, was “Have you had a fall in the past year?” [3,19]

#### Quality of Life

We evaluated participants’ quality of life (QoL) by using Short-Form 36 (SF-36), Taiwan version. This 36-item questionnaire measured 8 health concepts: Physical Functioning (10 items), Role Physical (4 items), Bodily Pain (2 items), General Health (5 items), Vitality (4 items), Social Functioning (2 items), Role Emotional (3 items), Mental Health (5 items) and Reported Health Transition (1 item) [40]. These 8 concepts could be grouped into Physical Component Summary (PCS) and Mental Component Summary (MCS) measures, which represent the subjective physical health and mental health respectively. We rated PCS and MCS according to the SF-36 users’ manual [41].

#### Demographics

The variables recorded were age, gender, marital status (categorized as married, single, divorced, widowed or separated), educational level (categorized as literate or illiterate), status of cigarette smoking (categorized as non-smoker, current smoker or ex-smoker), alcohol consumption (categorized as non-drinker, current drinker or ex-drinker), living status (categorized as living alone or not living alone), and accessibility of medical help in the event of an emergency, as the availability of social support in an emergency (categorized as could get help by himself/herself, could get help from relatives, could get help from public resources or could not get any help).

#### Medical Conditions, Insomnia, Sleep Quality, Depression and Subjective Health

Recorded medical conditions currently being treated included hypertension, cardiovascular diseases, diabetes mellitus, and stroke. Insomnia was identified by asking “Have you ever experienced insomnia more than twice in a week for more than 1 month?” Sleep quality was measured by the Pittsburgh Sleep Quality Index (PSQI) and a score > 5 indicated poor sleep quality [42]. Depressive symptoms were evaluated by the short form of the Geriatric Depression Scale (GDS-15). GDS-15 included 15 items with score ranges from 0 to 15, for which a score ≥ 5 represents possible clinical depression and a high score indicates severe depression. GDS-15 has been widely used in the community to evaluate the depressive symptoms in the elderly with good reliability [43,44]. The status of subjective health was measured by the first question of SF-36 [40].

### Statistical Analyses

Statistical analyses were performed by IBM SPSS version 20.0 (IBM Corporation, Armonk, NY, USA). For descriptive statistics, continuous variables were analyzed with Student’s *t* tests, while categorical variables were analyzed with chi-square tests. Multiple logistic regressions with forward stepwise methods were used to evaluate factors associated with FOF for all subjects. A two-tailed *P* value < 0.05 was considered statistically significant.

## Results

Two thousand one hundred and fifty-eight (56.4%) subjects were male and 1,666 (43.6%) subjects were female. The average ages were 73.9±5.8 years, 74.1±5.8 years and 73.5±5.8 years for all subjects, men and women, respectively. There were no significant differences between the subjects and the registered data from 1999 on the entire population aged ≥ 65 years throughout Taipei City, both for age (χ^2^ = 3.49, degree of freedom = 1, P = 0.06) and gender (χ^2^ = 2.01, degree of freedom = 1, P = 0.16). Among the 3,824 subjects enrolled in this study, 2,042 (53.4%) reported FOF, with FOF prevalence being 45.9% for men and 63.1% for women.

### QoL: SF-36 scores

The scores of 8 health concepts on SF-36, were significantly lower in subjects who reported FOF than in those without FOF, for male and female subjects (Table 1). The PCS and MCS scores for men and women with FOF were significantly lower than those without FOF (52.0±8.1 vs. 54.8±6.0, and 52.9±7.1 vs. 55.0±5.9 for men; 50.0±9.2 vs. 52.6±7.4 and 52.3±8.0 vs. 54.8±5.9 for women, respectively; all p < 0.01).

**Table 1:** Comparisons of SF-36 scores of subjects with and without fear of falling by different genders (Total n = 3824, including 2158 men and 1666 women).

	Fear of falling for men		P value for t test	Fear of falling for women		P value for t test
	No (n = 1167)	Yes (n = 991)		No (n = 615)	Yes (n = 1051)	
**SF-36 (mean, SD)**						
Physical Functioning	53.3±6.0	50.5±8.3	< 0.01	51.2±6.8	48.4±9.6	< 0.01
Role Physical	53.6±7.2	50.4±10.3	< 0.01	51.6±9.5	49.2±12.7	< 0.01
Bodily Pain	59.2±7.0	58.1±8.1	< 0.01	57.3±8.2	55.4v9.5	< 0.01
General Health	52.2±8.2	48.4±9.1	< 0.01	51.2±8.7	47.5±9.7	< 0.01
Vitality	60.0±6.7	57.3±7.0	< 0.01	58.9±6.8	55.9±7.7	< 0.01
Social Functioning	54.5±6.4	51.5±8.2	< 0.01	54.0±6.9	50.8±9.2	< 0.01
Role Emotional	54.0±6.0	52.7±8.0	< 0.01	53.7±6.6	52.4±11.2	< 0.01
Mental Health	53.6±7.4	51.0±8.2	< 0.01	52.5±7.2	49.2±8.6	< 0.01
**Summary scores**						
PCS	54.8±6.0	52.0±8.1	< 0.01	52.6±7.4	50.0±9.1	< 0.01
MCS	55.0±5.9	52.9±7.1	< 0.01	54.8±5.9	52.3±8.0	< 0.01

### Demographic Characteristics, History of Falls, Living Status, and Accessibility of Medical Help in the Event of an Emergency

For male subjects who reported FOF, the rate of those aged ≥ 75 years was significantly higher than subjects who had no FOF (54.0% vs. 46.0%, p < 0.01). Marital status, educational level, status of cigarette smoking, alcohol consumption and living status were not significantly different between subjects with FOF and those without (p = 0.38, 0.87, 0.44, 0.45 and 1.00, respectively). Subjects with FOF had a higher rate of falls than those without (64.6% vs. 35.4%, p < 0.01). The accessibility of medical help in the event of an emergency was significantly different between men with FOF and those without (p < 0.01). Fewer men with FOF could get help for themselves in an emergency than those without (40.3% vs. 59.7%). However, more men with FOF could not get any help than those without (53.7% vs. 46.3%; Table 2).

**Table 2:** Prevalence of fear of falling according to sociodemographic strata.

	Total subjects for men (n = 2,158)		Fear of falling for men (n = 991)		P value for Chi-square	Total subjects for women (n = 1,666)		Fear of falling for women (n = 1,051)		P value for Chi-square
Characteristics	n	%	n	Rate (%)	test	n	%	n	Rate (%)	test
**Age (years)**					< 0.01					< 0.01
65–74	1,286	59.6	520	40.4		1,007	60.4	593	58.9	
≥ 75	872	40.4	471	54.0		659	39.6	458	69.5	
**Falls in the past year**										
No	1,932	89.5	845	43.7	< 0.01	1,372	82.4	821	59.8	< 0.01
Yes	226	10.5	146	64.6		294	17.6	230	78.2	
**Living status**					0.94					0.06
Not living alone	2,052	95.1	942	45.9		1,560	93.6	975	62.5	
Living alone	106	4.9	49	46.2		106	6.4	76	71.7	
**Medical help accessibility in case of emergency**					< 0.01					0.16
Help by oneself	1,091	50.6	440	40.3		514	31.0	310	60.3	
Help from relatives	719	33.4	375	52.2		843	50.8	535	63.5	
Help from public resources	290	13.5	145	50.0		192	11.6	125	65.1	
Can't get any help	54	2.5	29	53.7		111	6.7	79	71.2	

For female subjects, the rate of those aged ≥ 75 years with FOF was significantly higher than those without (69.5% vs. 30.5%, p < 0.01). Marital status, educational level, status of cigarette smoking, alcohol consumption, living status and accessibility of medical help in the event of an emergency were not significantly different between women with FOF and those without (p = 0.35, 0.96, 0.38, 0.17, 0.06 and 0.16, respectively). Women with FOF had a higher rate of falls than those without (78.2% vs. 21.8%, p < 0.01) (Table 2).

### Medical Conditions, Clinical Characteristics, Sleep Quality, Depression and Subjective Health of Subjects

For medical conditions, both male and female subjects with FOF had higher rates of diabetes mellitus, cardiovascular diseases, stroke and insomnia than those without (p < 0.01, p = 0.02, p < 0.01 and p < 0.01 for men, respectively; p = 0.05, p < 0.01, p = 0.01 and p < 0.01 for women, respectively). Furthermore, a higher number of subjects with FOF had a PSQI > 5 than those without, both for male and female subjects (52.5% vs. 47.5% for men, and 70.6% vs. 29.4% for women, respectively; both p = 0.01), and more subjects with FOF had a GDS-15 score ≥ 5 than those without, for both male and female subjects (65.0% vs. 35.0% for men and 81.5% vs. 19.5% for women, respectively; both p < 0.01). For subjective health, subjects with FOF had poorer subjective health than those without, for both male and female subjects (both p < 0.01; Table 3).

**Table 3:** Prevalence of fear of falling according to clinical conditions.

	Total subjects for men (n = 2,158)		Fear of falling for men (n = 991)			Total subjects for women (n = 1,666)		Fear of falling for women (n = 1,051)		
Clinical conditions	n	%	n	Rate (%)	P value for Chi-square test	n	%	n	Rate (%)	P value for Chi-square test
**Medical conditions**										
Diabetes mellitus					< 0.01					0.05
No	1,871	86.7	830	44.4		1,408	84.5	876	62.2	
Yes	286	13.3	161	56.3		258	15.5	175	67.8	
Hypertension					< 0.01					0.05
No	1,271	58.9	548	43.1		955	57.3	583	61.0	
Yes	886	41.1	443	50.0		711	43.7	468	65.8	
Cardiovascular diseases					0.02					< 0.01
No	1,692	78.4	756	44.7		1,314	78.9	800	60.9	
Yes	465	21.6	235	50.5		352	21.1	251	71.3	
Stroke					< 0.01					0.01
No	2,053	95.2	920	44.8		1,616	97.0	1011	62.6	
Yes	104	4.8	71	68.3		50	3.0	40	80.0	
**Insomnia**					< 0.01					< 0.01
No	1,831	84.8	810	44.2		1,241	74.5	735	59.2	
Yes	327	15.2	181	55.4		425	25.5	316	74.4	
**Pittsburgh Sleep Quality Index**					0.01					0.01
≤5	1,830	84.8	819	44.8		1,302	78.2	794	61.0	
>5	328	15.2	172	52.4		364	21.8	257	70.6	
**GDS score**					< 0.01					< 0.01
<5	1,995	92.4	885	44.4		1,466	88.0	888	60.6	
≥5	163	7.6	106	65.0		200	12.0	163	81.5	
**Subjective health**					< 0.01					< 0.01
Excellent	47	2.2	16	34.0		20	1.2	7	35.0	
Very good	378	17.5	141	37.3		225	13.5	124	55.1	
Good	695	32.2	297	42.7		450	27.0	270	60.0	
Fair	911	42.2	450	49.4		851	51.1	546	64.2	
Poor	127	5.9	87	68.5		118	7.1	102	86.4	

### Multiple Logistic Regression Analysis of Factors Associated with FOF

For the full multiple logistic regression model for all subjects, the initial variables entered into the model were: falls in the past year; demographic characteristics including age, gender, marital status and education level; personal characteristics including smoking and alcohol consumption, living status and accessibility of medical help in the event of an emergency; chronic conditions including diabetes mellitus, hypertension, cardiovascular diseases and stroke; insomnia; sleep quality by PSQI score; GDS score and subjective health. This model revealed that significant associated factors for FOF among all subjects included falls in the past year (odds ratio [OR] = 2.23, 95% confidence interval [95% CI] = 1.80–2.76), age ≥ 75 years (OR:1.52, 95% CI:1.32–1.75), being female (OR:1.78, 95% CI:1.55–2.05), help from relatives (vs. help by oneself, OR:1.32, 95% CI:1.14–1.62), help from public resources (vs. help by oneself, OR:1.28, 95% CI:1.03–1.58), diabetes mellitus (OR:1.32, 95% CI:1.09–1.62), cardiovascular diseases (OR:1.19, 95% CI:1.00–1.41), stroke (OR:1.94, 95% CI:1.33–2.83), insomnia (OR:1.50, 95% CI:1.26–1.80), a GDS score ≥ 5 (vs. GDS score < 5, OR:1.78, 95% CI:1.34–2.33), good and fair subjective health (vs. excellent and very good subjective health, OR:1.35, 95% CI:1.13–1.62) and poor subjective health (vs. excellent and very good subjective health, OR:2.52, 95% CI:1.75–3.64).

The associated factors for FOF among male subjects included falls in the past year (OR:2.24, 95% CI:1.66–3.03), age ≥ 75 years (OR:1.60, 95% CI: 1.33–1.92), help from relatives (vs. help by oneself, OR:1.49, 95% CI: 1.22–1.82), help from public resources (vs. help by oneself, OR:1.37, 95% CI:1.05–1.80), diabetes mellitus (OR:1.56, 95% CI: 1.20–1.78), stroke (OR:2.06, 95% CI:1.32–3.20), insomnia (OR:1.38, 95% CI:1.07–1.78), a GDS score ≥ 5 (vs. GDS score < 5, OR:1.67, 95% CI:1.15–2.42), good and fair subjective health (vs. excellent and very good subjective health, OR:1.40, 95% CI: 1.11–1.76) and poor subjective health (vs. excellent and very good subjective health, OR:2.44, 95% CI: 1.54–3.86).

The associated factors for FOF among female subjects included falls in the past year (OR: 2.19, 95% CI: 1.61–2.97), age ≥ 75 years (OR: 1.42, 95% CI: 1.14–1.76), cardiovascular diseases (OR:1.32, 95% CI: 1.00–1.73), insomnia (OR:1.65, 95% CI:1.27–2.13), a GDS score ≥ 5 (vs. GDS score < 5, OR:2.05, 95% CI:1.38–3.04), good and fair subjective health (vs. excellent and very good subjective health, OR:1.32, 95% CI: 1.00–1.75) and poor subjective health (vs. excellent and very good subjective health, OR:3.12, 95% CI:1.69–5.76) (Table 4).

**Table 4:** Factors associated with fear of falling by stepwise logistic regression.

	Model 1^a^		Model 2^a^ (Men)		Model 3^a^ (Women)	
Variables	Odds Ratio	95% CI	Odds Ratio	95% CI	Odds Ratio	95% CI
**Falls in the past year**						
No	reference		reference		reference	
Yes	2.23	1.80–2.76	2.24	1.66–3.03	2.19	1.61–2.97
**Age(years)**						
65–74	reference		reference		reference	
≥75	1.52	1.32–1.75	1.60	1.33–1.92	1.42	1.14–1.76
**Sex**						
Male	reference		-	-	-	-
Female	1.78	1.55–2.05	-	-	-	-
**Medical help accessibility in case of emergency**						
Help by oneself	reference		reference		-	-
Help from relatives	1.32	1.14–1.62	1.49	1.22–1.82	-	-
Help from public resources	1.28	1.03–1.59	1.37	1.05–1.80	-	-
Can't get any help	1.38	0.97–1.99	1.17	0.65–2.10	-	-
**Chronic conditions**						
Diabetes mellitus						
No	reference		reference		-	-
Yes	1.32	1.09–1.62	1.56	1.20–1.78	-	-
Hypertension						
No	-	-	-	-	-	-
Yes	-	-	-	-	-	-
Cardiovascular diseases						
No	reference		-	-	reference	-
Yes	1.19	1.00–1.41	-	-	1.32	1.00–1.73
Stroke						
No	reference		reference		-	-
Yes	1.94	1.33–2.83	2.06	1.32–3.20	-	-
**Insomnia**						
No	reference		reference		reference	
Yes	1.50	1.26–1.80	1.38	1.07–1.78	1.65	1.27–2.13
**Depression**						
GDS score< 5	reference		reference		reference	
GDS score≥ 5	1.78	1.34–2.33	1.67	1.15–2.42	2.05	1.38–3.04
**Subjective health**						
Excellent and very good	reference		reference		reference	
Good and fair	1.35	1.13–1.62	1.40	1.11–1.76	1.32	1.00–1.75
Poor	2.52	1.75–3.64	2.44	1.54–3.86	3.12	1.69–5.76
Cox & Snell R Square	0.10		0.075		0.072	

^a^Initial variables entered in the model were: fall in the past year; demographic characteristics including age, gender, marital status, education level; personal characteristics including smoking and alcoholic drinking, living status, medical help accessibility in case of emergency; chronic conditions including diabetes mellitus, hypertension, cardiovascular diseases and stroke; insomnia; sleep quality by Pittsburgh Sleep Quality Index (PSQI) score; Geriatric Depression Scale (GDS) score and subjective health.

## Discussion

This study reported the quality of life and associated factors with FOF for older adults living in the community. The overall prevalence of FOF in this study was 53.4%. Subjects with FOF had lower scores for SF-36 in 8 concepts, and therefore lower PCS and MCS scores, than non-FOF subjects, both for male and female subjects. Female subjects with FOF had a worse score on SF-36 than male subjects with FOF. Falls in the previous year, older age, accessibility of medical help in the event of an emergency, diabetes mellitus, stroke, insomnia, depression and worse subjective health were associated factors of FOF for male subjects. For female subjects, falls in the previous year, older age, cardiovascular diseases, insomnia, depression and worse subjective health were associated factors of FOF. Associated factors of FOF that differed for men and women were accessibility of medical help in the event of an emergency, diabetes mellitus and stroke, and cardiovascular diseases.

The overall prevalence of FOF for all subjects in the current study was 53.4%, which was similar to reports in several previous studies [4–6] but lower than a recent study in Korea [10]. The difference in prevalence might be related to study area, subjects’ gender and age distributions, and FOF measurements between studies. In this study, the prevalence of FOF was 45.9% for men and 63.1% for women. One reason for this gender difference found in this study might be that women are more likely to fall than men, and the other reason might be that more women have persistent FOF than men [23,28].

In the present study, common associated factors of FOF for both gender, namely falls in the previous year, older age, insomnia, depression and worse subjective health were found. A history of falls [4,7,9], older age [1,2,4], depression [7,9,22] and worse subjective health [2,4] have been widely reported to be risk factors for FOF in previous studies. Insomnia as a novel associated factor for FOF, noted in this study, may have several causes. First, inappropriate hypnotics use for insomnia may be related to dizziness, an unsteady gait and weakness. Second, chronic conditions such as chronic obstructive pulmonary diseases, heart failure, and strokes and may be related to insomnia, and these conditions may be related to poor physical condition and frailty. Third, some affective disorders may be related to insomnia, and psychoactive medications in combination with the use of hypnotics may aggravate dizziness, unsteady gait and weakness. However, we have controlled for confounding effects from depression and various medical conditions in the present study. Therefore, insomnia might be an independent factor of FOF in older adults.

With regards to the differential correlates for FOF in both gender, we found diabetes mellitus and stroke were risk factors for FOF in men, while cardiovascular diseases were a risk factor for FOF in women. A recent study reported that subjects with diabetes had worse mobility and were associated with an increased FOF and fear-associated activity restriction [45]. Another study found that 50% of subacute hemiplegic stroke patients had FOF, which was related to muscle strength issues with their lower limbs [46]. A previous study revealed that FOF was noted in claudicated patients who had poor physical, social, and psychological functioning [47]. In this study, we found that the accessibility of medical help in the event of an emergency was associated with FOF in men, but not in women. The accessibility of medical help in the event of an emergency is related to an individual’s social support system and, in our case, may be related to the degree of independence. We found that men had a relatively low rate of seeking help in an emergency compared to women. This may be related to different help-seeking behaviors between different genders. Previous studies reported that men were less likely to seek health-related help and use mental and physical health resources [48]. The reasons for gender differences are complex, but may include gender roles, masculinity and other factors that may cause men to be less prone to seek help than women are [49].

Several limitations of this study should be considered when interpreting the study results. First, the current study was a cross-sectional study which could only present the associations between risk factors and FOF, but not the causal relationships. Second, information about previously reported factors associated with FOF, including physical performance, anxiety, cognitive status and pain, was not sufficient. Nevertheless, lack of above-mentioned information might cause an underestimation of our results in this study. However, under the underestimated condition, we still found a significant association between accessibility of medical help and fear of falling in male subjects. The strength of our study was that it contains information about chronic conditions such as diabetes mellitus, hypertension, cardiovascular diseases, stroke, insomnia and depression, and the accessibility of medical help in the event of an emergency.

## Conclusions

FOF is a prevalent condition among older people living in the community. In addition to the well-known associated factors of FOF, insomnia is also a significant factor for FOF both in men and women. There are gender-specific associated factors of FOF in community-dwelling older adults, but the underlying mechanisms need further investigation. Furthermore, gender differences should be considered when planning prevention and intervention strategies for fear of falling among older people living in the community.

## Supporting Information

S1 Dataset (XLSX)

S1 Metafile (XLSX)
